# Ultralong lifetime and efficient room temperature phosphorescent carbon dots through multi-confinement structure design

**DOI:** 10.1038/s41467-020-19422-4

**Published:** 2020-11-05

**Authors:** Yuqiong Sun, Shuting Liu, Luyi Sun, Shuangshuang Wu, Guangqi Hu, Xiaoliang Pang, Andrew T. Smith, Chaofan Hu, Songshan Zeng, Weixing Wang, Yingliang Liu, Mingtao Zheng

**Affiliations:** 1 Key Laboratory for Biobased Materials and Energy of Ministry of Education/Guangdong Provincial Engineering Technology Research Center for Optical Agriculture, College of Materials and Energy, South China Agricultural University, Guangzhou, 510642 China; 2 Maoming Branch, Guangdong Laboratory for Lingnan Modern Agriculture, Maoming, 525000 China; 3 Polymer Program, Institute of Materials Science and Department of Chemical & Biomolecular Engineering, University of Connecticut, Storrs, CT 06269 USA; 4 Ministry of Education Key Laboratory of Enhanced Heat Transfer & Energy Conservation, South China University of Technology, Guangzhou, 510640 China

**Keywords:** Optical materials, Nanoparticles

## Abstract

Room temperature phosphorescence materials have inspired extensive attention owing to their great potential in optical applications. However, it is hard to achieve a room temperature phosphorescence material with simultaneous long lifetime and high phosphorescence quantum efficiency. Herein, multi-confined carbon dots were designed and fabricated, enabling room temperature phosphorescence material with simultaneous ultralong lifetime, high phosphorescence quantum efficiency, and excellent stability. The multi-confinement by a highly rigid network, stable covalent bonding, and 3D spatial restriction efficiently rigidified the triplet excited states of carbon dots from non-radiative deactivation. The as-designed multi-confined carbon dots exhibit ultralong lifetime of 5.72 s, phosphorescence quantum efficiency of 26.36%, and exceptional stability against strong oxidants, acids and bases, as well as polar solvents. This work provides design principles and a universal strategy to construct metal-free room temperature phosphorescence materials with ultralong lifetime, high phosphorescence quantum efficiency, and high stability for promising applications, especially under harsh conditions.

## Abstract (web summary)

For room temperature phosphorescence (RTP) materials to reach their potential for optical applications, new materials with improved performance must be realized. Here, the authors report multi-confined carbon dots as high stability RTP materials with long afterglow lifetime & high efficiency.

## Introduction

Room temperature phosphorescence (RTP) materials, as a typical class of long-lived emission materials, have evoked considerable attention during the past decade thanks to their promising applications in displays, chemical sensors, bioimaging, and anti-counterfeiting^1^. The present long-lived luminescent materials are mainly organic compounds through molecular design and inorganic complexes which are typically designed based on transition metals and rare-earth ions. Attractive RTP organics, which may enable conventional phosphorescence through the formation of specific structure (i.e., H-aggregation, host–guest composition and polymer matrix)^2–5^ to stabilize the triplet excitations or introducing special moieties (i.e., heavy halogen and aromatic aldehyde)^6,7^ to enhance the intersystem crossing (Supplementary Fig. 1a). Nevertheless, the current organic RTP materials present short afterglow lifetime of several milliseconds, low stability, extreme sensitivity to oxygen/vapor, and toxicity that cannot be neglected, which significantly limit their potential applications^8–10^. In contrast, traditional inorganic compounds exhibit superior long lifetime due to the unique exciton transition process, of which the emission is caused by slow release of trapped charge (traps formed by structure defects or complex impurities) by the thermal disturbance of ambient temperature (Supplementary Fig. 1b)^11,12^. However, given the scarcity of metal resources, complicated fabrication processes, and extreme instability in humid environments, none of these materials are ideal for permanent considerations. Especially, it is very difficult to simultaneously obtain an efficient RTP material with long afterglow lifetime and high quantum efficiency^10,13^. More recently, great efforts have been devoted to increasing quantum efficiency and prolonging lifetime of RTP materials by molecular design^8,10,13–15^. For instance, An’s group made significant achievements to simultaneously enhance efficiency and lifetime of organic phosphorescence by molecular self-assembly^8,10^. Tang’s group made remarkable progress on developing persistent and efficient pure organic RTP materials by intrinsic molecular-structure engineering or intramolecular triplet–triplet energy transfer^13–15^. However, current RTP materials still suffer from the short afterglow lifetime, low phosphorescence quantum efficiency (PQE), and poor stability. Therefore, exploring new generation of RTP materials with simultaneous long lifetime, high efficiency, and excellent stability through facile, green, and cost-effective methodology is highly desirable but remains a formidable challenge.

**Fig. 1:**
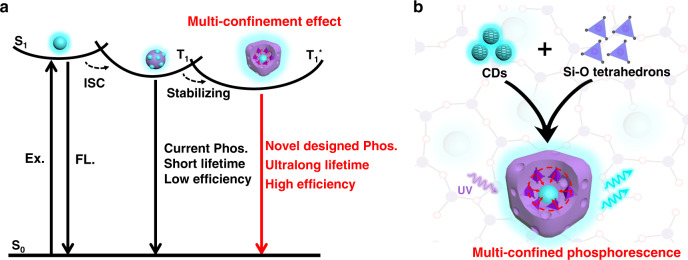
Schematic of the proposed phosphorescence mechanism and design strategy of multi-confined CDs@SiO_2_ RTP material. **a** Proposed mechanism of CDs in conventional matrix and multi-confined structure. (S0 ground state, S1 first excited singlet state, T1 first excited triplet state, ISC intersystem crossing, Ex. excitation, FL. fluorescence, Phos. phosphorescence). **b** Schematic of the design strategy of the multi-confined (rigid network, stable covalent bonds, and three-dimensional nano-space) phosphorescence.

In the past decade, photoluminescence (PL) carbon dots (CDs) have triggered tremendous attention because of their facile preparation, high photo-stability, and low-toxicity^16^. Generally, CDs do not exhibit phosphorescence due to their unstable triplet excited states^17,18^. Great effort has been devoted to stabilizing triplet excited states of CDs through constructing hybrid systems of CDs in organic or inorganic matrices in order to achieve RTP^17–21^. Auxiliary matrices, including inorganic complexes (e.g., KAl(SO_4_)_2_·*x*H_2_O, SiO_2_, zeolites, layered double hydroxides (LDHs), etc.)^17–19,21–25^ and polymers/organic compounds (e.g., poly(vinyl alcohol), polyurethane, cyanuric acid, urea, etc.)^26–29^, were exploited to fabricate a matrix-assisted isolation structure. These substrates enable CDs to generate typical RTP emission by suppressing the intermolecular vibration (left in Fig. 1a)^30,31^. Unfortunately, current CD-based RTP materials generally face common troubles, including but not limited to, relatively short lifetime, low quantum efficiency, and poor stability, not to mention possess long lifetime and high efficiency simultaneously. Currently, there are three strategies to rigidify the triplets of CDs: (1) hydrogen bonds^20^. Early on researchers incorporated CDs into various matrices such as PVA to achieve RTP, in which hydrogen bonds formed between the CDs and the PVA matrix can protect the triplet excited states of CDs from rotational or vibrational loss. However, the strength of hydrogen bonds is relatively weak, thereby their effect on stabilizing triplet states is limited. (2) Covalent bonds^20^. Compared to weak hydrogen bonds, covalent bonds can be employed as a much more effective alternative to fix and rigidify triplet emission species. Therefore, researchers tried to incorporate CDs into colloidal silica through covalent bonds, and some significant features such as long afterglow and aqueous-related afterglow were observed^17,22^, since the emissive moieties could be fixed on certain substances by covalent bonds. However, in previous reports, the carbon and silicon used are usually derived from separate sources, and the recombination of the CDs with the silica matrices are generally carried out at low temperatures, which makes it difficult to form very stable C–Si bonds. (3) Structural confinements^18,19,24^. With further research progress, it was found that the incorporation of CDs into zeolites or two-dimensional LDHs can generate a structural confinement effect on CDs. However, CDs can only be embedded in the interrupted nano-space or attach to the external surface of zeolite host due to the larger size of CDs (≈2–6 nm) compared to the diameter of micropores (less than 2 nm)^19^. Therefore, zeolites can only exert a relatively weak surface confinement to the embedded CDs, which is similar to the confinement effect by two-dimensional interlayer of LDHs, resulting in relatively short lifetime and low quantum efficiency. Generally, the phosphorescence lifetime of the reported CD-based RTP materials are mostly less than 2 s, accompanied with a PQE less than 20%^32^. In brief, the early work has shown that simply relying on one single confinement effect is not enough to achieve the desired RTP performance; breakthroughs on the phosphorescence lifetime, quantum efficiency, and stability of the CD-based RTP materials have not yet been made. Thus, exploration of design principles and fabrication strategies to effectively rigidify the triplet excited states of CDs for achieving a long-lived and efficient metal-free RTP material is highly desired but greatly challenging.

To achieve efficient RTP of CDs, some key features and principles must be satisfied in the fabrication strategy. Firstly, it is essential to construct a highly rigid network that can embed and isolate CDs from external quenching factors (such as oxygen, humidity, etc.). Secondly, it is vital to form stable covalent bonds between the matrix network and the embedded CDs to minimize the non-radiative decay of long-lived triplets. Thirdly, it is crucial to tightly fix the CDs in three-dimensional (3D) nano-space to produce an efficient spatial restriction, so as to effectively suppress the intramolecular vibration and stabilize the triplet excited states of CDs.

In this work, a strategy is designed and exploited to fabricate metal-free multi-confined CDs within SiO_2_ (CDs@SiO_2_) with ultralong RTP lifetime, high PQE, and exceptional stability simultaneously, by constructing an effective multi-confinement effect (MCE). Our results demonstrate that this unique multi-confinement exerted by tetrahedral interstice is more effective than conventional surface confinement of zeolites and LDHs^18,24^. It is also different from the ones covalently linked to a colloidal silica derived from the hydrolysis of tetraethyl orthosilicate^17^. The as-designed multi-confined RTP of CDs@SiO_2_ not only exhibits an ultralong RTP lifetime of 5.72 s (more than 40 s to the naked eye), but also achieves a very high PQE of 21.30%. Moreover, the as-fabricated CDs@SiO_2_ phosphors exhibit high stability, which can resist the quenching from strong oxidants, strong acids, and bases, water, as well as polar solvents. More importantly, this method can be easily expanded to other silicon-rich biomasses and other regular chemicals (instead of restricting to biomass only) to ensure a reproducible production of high quality multi-confined RTP materials with a potential for future large-scale manufacturing. Therefore, this work provides a universal strategy to fabricate CDs@SiO_2_ phosphors and offers design principles and insights into metal-free RTP materials with ultralong lifetime, high quantum efficiency, and high stability simultaneously for various RTP-based applications.

## Results

### Design of multi-confined CDs@SiO_2_

To achieve the above outlined principles, a strategy was developed to synthesize metal-free multi-confined RTP CDs@SiO_2_ by constructing an effective MCE. First, rice husks (RHs) with a homogeneous distribution of carbon and silicon were employed as both the carbon and silicon source. By taking advantage of the intimate contact of carbon and silica in RHs^33^, we designed and achieved stable covalent interactions between the CDs and the SiO_2_ matrix. Second, a sol–gel process was developed to fabricate a vast H_4_SiO_4_ network linked by Si–O–Si chains. Note that the luminescent CDs were in situ generated and perfectly fixed within the polymerized network. Third, the subsequent high temperature calcination process not only results in a conversion of loose H_4_SiO_4_ to a rigid and compact silica network, but also forms stable covalent interaction between the CDs and the silica network, leading to the formation of multi-confined CDs. As depicted in Fig. 1a, b, CDs were in situ embedded into a vast SiO_2_ network composed of Si–O tetrahedrons. Appropriate 3D nano-space surrounded by Si–O tetrahedrons can tightly accommodate and embed CDs. The MCE consists of a highly rigid network, stable covalent bonding, and 3D spatial restriction that can effectively suppress the intramolecular vibration and thus successfully stabilizing the triplet excited states of the CDs.

### Synthesis and characterization of CDs@SiO_2_

**Fig. 2:**
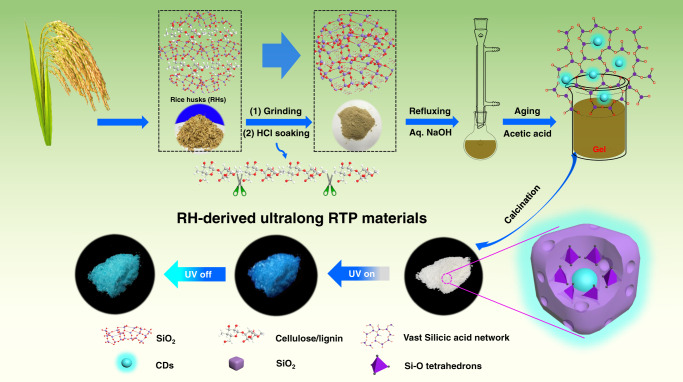
Design of multi-confined CDs@SiO_2_. Schematic of the overall process for fabrication of the metal-free CDs@SiO_2_ RTP materials with ultralong lifetime from rice husks (RHs).

RHs are often treated as a biowaste, converting RHs biowaste into value-added products with broad applications remains a considerable challenge as well as a significant task^33,34^. In this work, we take advantage of the intimate contact of C and silica in RHs to design and fabricate CDs@SiO_2_ network with ultralong RTP lifetime, remarkable quantum efficiency, and extraordinary stability through an in situ method. As illustrated in Fig. 2, during the reflux pretreatment process, the SiO_2_ in RHs first reacted with NaOH to form Na_2_SiO_3_, as shown in Eq. 1. Simultaneously, some carbonaceous organics such as cellulose in RHs can be transferred into PL CDs at a temperature of 160 °C^35,36^. The as-generated Na_2_SiO_3_ was then turned into mono-silicic acid (H_4_SiO_4_) in an acidic environment (Eq. 2). The freshly generated H_4_SiO_4_ can be polymerized to form a vast network linked by Si–O–Si chains during the aging process^25^, which plays a crucial role in the whole preparation process, as shown in Eq. 3. Note that the luminescent CDs could be perfectly fixed inside the polymerized H_4_SiO_4_ network that protects the CDs from calcination at high temperature, forming spatially confined CDs@SiO_2_ phosphors with ultralong RTP and high stability. In order to optimize RTP luminescence, a series of experiments were conducted to fabricate CDs@SiO_2_-*x* (*x* refers to the calcination temperature) phosphors at various calcination temperatures. As shown in Supplementary Figs. 2 and 3 and Supplementary Table 1, the resultant CDs@SiO_2_-600 phosphors possess the ultralong phosphorescent lifetime and high quantum efficiency.

1 $${\mathrm{SiO}}_2 + 2{\mathrm{NaOH}} \to {\mathrm{Na}}_2{\mathrm{SiO}}_3 + {\mathrm{H}}_2{\mathrm{O}}$$

2 $${\mathrm{Na}}_{\mathrm{2}}{\mathrm{SiO}}_3 + 2{\mathrm{HAc}} + {\mathrm{H}}_2{\mathrm{O}} \to {\mathrm{H}}_4{\mathrm{SiO}}_4 + 2{\mathrm{NaAc}}$$

3

**Fig. 3:**
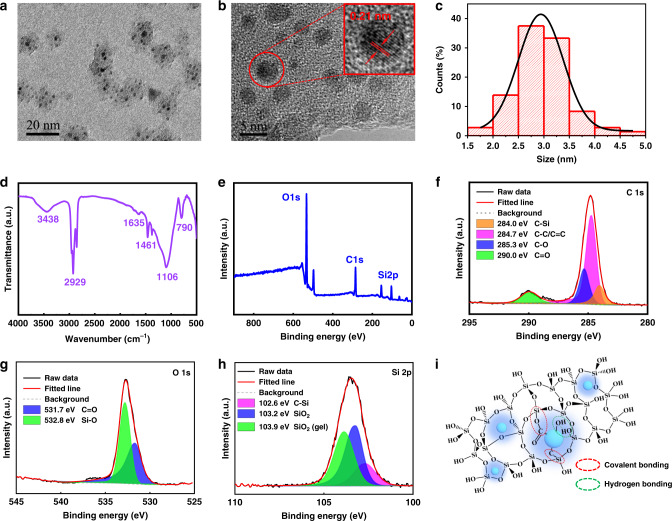
Morphology, FTIR, and XPS characterization of the CDs@SiO_2_. **a** TEM and **b** HRTEM images of CDs@SiO_2_-600. **c** Size distribution histogram of the CDs in SiO_2_ matrix prepared at 600 °C. **d** FTIR spectrum and **e** XPS survey spectrum of CDs@SiO_2_-600. **f**–**h** High resolution XPS spectrum and fitting results of C 1s (**f**), O 1s (**g**), and Si 2p (**h**) in CDs@SiO_2_-600. **i** Schematic of the proposed structure of CDs@SiO_2_.

As shown in Fig. 3a, b and Supplementary Fig. 4, the transmission electron microscopy (TEM), high-resolution TEM (HRTEM) images, and X-ray diffraction (XRD) pattern of CDs@SiO_2_-600 reveal the existence of amorphous silica phase, that is the random network of Si–O tetrahedrons^37^, and the even distribution of CDs in the amorphous SiO_2_ network. The HRTEM image displayed in Fig. 3b shows that the CDs embedded in SiO_2_ exhibit an average diameter of 3.0 ± 0.5 nm (Fig. 3c) and a lattice fringe with a spacing of 0.21 nm (Fig. 3b inset). Note that the pristine CDs derived from the mother liquor, through a dialysis, possess an average size of 4.8 nm (Supplementary Fig. 5a, b). The decrease of the particle size of the CDs might be due to the reduction of surface groups during the calcination process^25^.

To further deduce the chemical interactions between the CDs and the SiO_2_, Fourier transform-infrared spectroscopy (FTIR) and X-ray photoelectron spectroscopy (XPS) of CDs and CDs@SiO_2_-600 were carried out. The FTIR spectrum (Fig. 3d) shows characteristic peaks at 1635 and 3438 cm^−1^ that can be attributed to C = O and O–H stretching vibrations, respectively. Importantly, the obvious peaks around 1106 cm^−1^ (Si–O–C stretching vibrations) and 790 cm^−1^ (Si–C stretching vibrations) reveal the formation of covalent bonds between the CDs and the amorphous SiO_2_, suggesting that the CDs and the Si–O network are connected by covalent bonds. The XPS results further confirm this hypothesis. The XPS survey scan in Fig. 3e shows that this nanocomposite mainly consists of C, O, and Si elements. The XPS C 1s spectra (Fig. 3f) indicated the presence of C–Si (284.0 eV), C–C/C = C (284.7 eV), C–O (285.3 eV), and C = O (290.0 eV) bonds^22,28^. The XPS O 1 s spectra (Fig. 3g) shows two peaks at 531.7 and 532.8 eV that can be assigned to C = O and Si–O bonds, respectively^25^. The XPS Si 2p spectra (Fig. 3h) exhibits three binding energies at 102.6, 103.2, and 103.9 eV, indicating the presence of Si–C, SiO_2_, and SiO_2_ (gel), respectively^22^. The existence of Si–C bonds was also verified in the samples calcined at other temperatures (Supplementary Fig. 6). Based on the above results, the structure and possible bonding between the CDs and the SiO_2_ network are illustrated in Fig. 3i. The initially synthesized CDs as luminescence centers with C = O and O–H functional groups (Supplementary Figs. 7 and 8) were embedded into the SiO_2_ matrix through both covalent and hydrogen bonds. Because of those bond interactions, the intermolecular motion of the CDs is prohibited and the long-lived phosphorescence occurs. Furthermore, this stable structure of CDs@SiO_2_ helps to suppress the non-radiative transition and makes it resistant to the quenching of strong oxidants, acids and bases, and polar solvents.

### Optical properties of CDs@SiO_2_

**Fig. 4:**
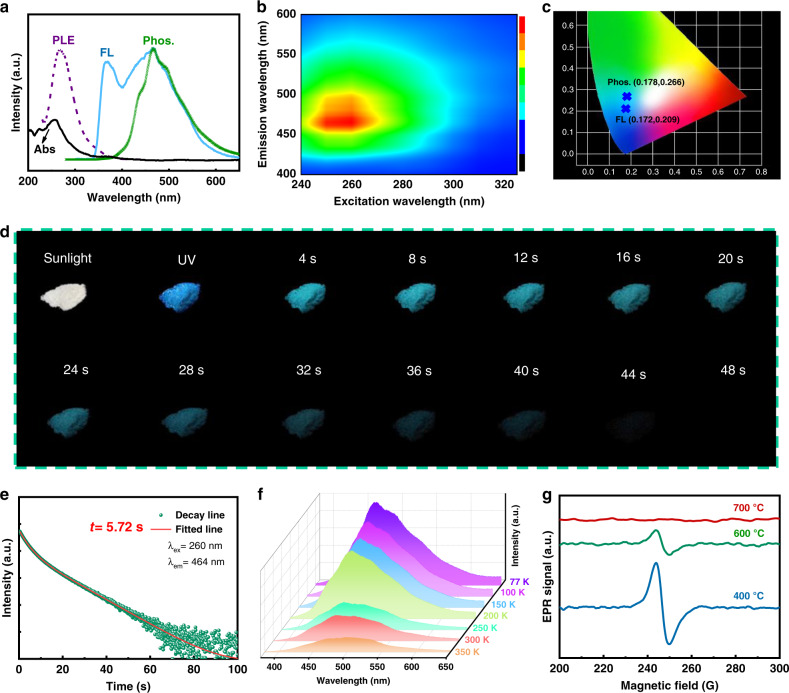
Optical properties of CDs@SiO_2_. **a** Absorption, photoluminescence excitation, fluorescence emission, and phosphorescence emission spectra of CDs@SiO_2_-600. **b** Phosphorescent two-dimensional excitation-emission plot of CDs@SiO_2_-600. Emission intensity rises with the color changing from blue to green and to red. **c** CIE coordinates of the fluorescence emission and phosphorescence of CDs@SiO_2_-600. **d** Photographs of CDs@SiO_2_-600 powders under sunlight, excited with 254 nm UV lamp, and after switching off UV. **e** Time-resolved phosphorescence decay and fitting curve (red line) of the emission bands at 464 nm with 260 nm excitation. **f** Temperature-dependent time-resolved phosphorescence spectra of CDs@SiO_2_-600. **g** EPR spectra of CDs@SiO_2_-400, CDs@SiO_2_-600, and CDs@SiO_2_-700.

The UV–vis absorption spectrum of CDs@SiO_2_-600 shown in Fig. 4a displays an absorption peak at 225 nm and a broad band from 240 to 370 nm, which are attributed to the π–π* transition of C = C and n–π* transition of C = O, respectively^24^. The PL excitation spectrum shows a broad band from 234 to 372 nm with the maximum excitation peaking at 260 nm, highly overlapping with the absorption band of C = O bonds, suggesting that the luminescence should come from the C = O bonds of CDs. The as-fabricated multi-confined RTP CDs@SiO_2_-600 presents blue fluorescence emission and ultralong phosphorescence at the excitation wavelength of 260 nm (Fig. 4a). As shown in the phosphorescent two-dimensional excitation-emission plot (Fig. 4b), the phosphorescence can be excited by wavelengths from 240 to 300 nm, with the best RTP emission obtained under 260 nm excitation centered at 464 nm. The best excitation wavelength of CDs@SiO_2_-600 is different from that of the pristine RH-derived CDs (Supplementary Fig. 9), indicating a blue shifting of excitation wavelength when CDs is embedded and restricted in silica matrix. Furthermore, the emission intensity rises with the color changing from blue to red, and the emission peaks show excitation wavelength independence. The color coordinates of the FL and phosphorescence correspond to (0.178, 0.266) and (0.172, 0.209), respectively, through the CIE (Commission International d’Éclairage) 1931 chromaticity coordinates (Fig. 4c).

The multi-confined RTP CDs@SiO_2_-600 exhibits intense blue-greenish RTP after switching off the 254 nm irradiation, which could last more than 40 s to the naked eye (Fig. 4d and Supplementary Movie 1). To obtain insights into the multi-confined RTP properties of the CDs@SiO_2_-600, the phosphorescence decay spectra was further measured (Fig. 4e), which could be well fitted to a tri-exponential function. According to the following equation:

4 $$\tau _{\rm{avg}} = {\sum} {\alpha _i\tau _i^2} /{\sum} {\alpha _i\tau _i},$$

the calculated average lifetime is up to 5.72 s. This is by far much higher than that of the previously reported CD-based RTP materials (Supplementary Table 2)^18–32,38–43^ and pure organic RTP materials^1–15^. The Δ*E*_ST_ between the lowest triplet (T_1_) and the lowest singlet (S_1_) gap was calculated to be 0.155 eV (S_1_ = 2.827 eV and T_1_ = 2.672 eV). Such a low value benefits effective ISC process to populate triplet excitations.

The temperature-dependent phosphorescence emission and lifetime decay were further investigated (Fig. 4f and Supplementary Fig. 10). The RTP intensity and lifetime of CDs@SiO_2_-600 apparently decreased as the temperature increased due to the slow radiative decay of long-lived excited states and the non-radiation transition that is largely activated at elevated temperatures^2,43,44^, suggesting a phosphorescence characteristic rather than delayed fluorescence. Meanwhile, the full width at half-maximum (FWHM) of the emission spectra generally broadens due to the thermal expansion and electron-phonon interactions^45^. According to the temperature dependence phosphorescence emission, the FWHM was extended from 103 to 116 nm when the temperature increases from 77 to 350 K. Such a slight change may be ascribed to the protection of MCE. It is worth mentioning that an ultralong phosphorescence lifetime (up to 3.43 s) was achieved when the temperature was raised to 350 K, which is still much higher than the previously reported state-of-the-art RTP materials (Supplementary Table 3). The understanding gained from the experimental results will help promote applications of the prepared CDs@SiO_2_ phosphors in high-temperature environment.

The emission from CDs@SiO_2_ phosphors is hypothesized to originate from carbon luminescence centers immobilized in the Si–O network, and the emission intensity also corresponds to the CDs content^46^. The carbonyl related groups on CDs can significantly improve the spin–orbit coupling to generate stable triplet excitons (unpaired electrons), thus resulting in phosphorescence emission. To verify our hypothesis, EPR spectra of the samples prepared at different calcination temperatures were recorded to characterize the stable triplet states. As shown in Fig. 4g, the intensity of the EPR signal at 240–260 G decreases with an increasing annealing temperature, indicating that the amount of luminescence centers gradually decreases with the increase of annealing temperature. Because the CDs are confined in the interstices between Si-O tetrahedrons, the spatial protection and restriction effect of the network protects the CDs from damage during the calcination process within an optimum temperature range. It is worth mentioning that the large numbers of carbon constituents in Si–O network calcined at low temperatures (e.g., 400 °C) are not beneficial to the luminescence. First, the high content of CDs might cause the high concentration annihilation characteristic. Moreover, we propose that the phosphorescence performance of CDs@SiO_2_ was not only influenced by the content of CDs, but largely affected by the multi-confinement structure. Although CDs@SiO_2_-400 had the largest numbers of luminescence centers, the weak spatial confinement caused by the loose Si–O network renders them to exhibit relatively poor phosphorescence performance. Meanwhile, if the calcination temperature is too high (700 °C), less luminescence centers are left, which also results in weaker emission^47^. The TGA curve of the gel can further verify the change of carbon contents (Supplementary Fig. 11). The weight of the gel reduced ca. 7.8% when the temperature was continuously increased from 400 to 600 °C, and then further decreased by 1.1% up to 700 °C. Therefore, a suitable calcination temperature (600 °C) is important to obtain excellent ultralong-lived RTP materials.

According to the above results, we propose that such an ultralong RTP is simultaneously attributed to the MCE of the interstices between Si and O tetrahedrons, which can not only anchor CDs to provide unique stability, but also exert an effective spatial restriction to suppress the intramolecular vibration and rigidify the triplet excited state of the CDs, as illustrated in Supplementary Fig. 12. It is noteworthy that high temperatures over 700 °C will lead to the collapse of these nano-space surrounded by Si–O tetrahedrons, resulting in the weakening of the MCE. The sample calcined at 300 °C, exhibiting no phosphorescence, also further verifying the structural state of the CDs@SiO_2_. But when recalcined at higher temperatures, the ultralong RTP was achieved again. This phenomenon suggests that the loose Si–O network cannot confine CDs well, but with an increasing calcination temperature, the loose structure converts into a compact short-range ordered state, which is necessary to exert a spatial restriction to the embedded CDs, as illustrated in Supplementary Fig. 12. Another evidence is that the XRD pattern shows that the CDs@SiO_2_ changes from amorphous to crystalline as the calcination temperature increases, which further indicates the formation of short-range ordered transition state between the amorphous and crystalline (Supplementary Fig. 3). To further highlight the MCE in multi-confined RTP CDs@SiO_2_, a CDs/n-SiO_2_ nanocomposite was prepared by mixing nano silica sol (n-SiO_2_) with RH-derived CDs directly and then subjected to the same calcination treatment. As shown in Supplementary Fig. 13, the CDs/n-SiO_2_ nanocomposite exhibits very weak phosphorescence that is barely captured because the CDs cannot be well embedded within the network structure of the pre-synthesized silica. These results suggest that the MCE of Si–O network is crucial to obtain ultralong phosphorescence.

Furthermore, the resultant CDs@SiO_2_ phosphors present very high absolute PQE of over 21% with ultralong lifetime (up to 5.72 s) simultaneously. The maximum absolute PQE of the resultant multi-confined RTP CDs@SiO_2_ reached 26.36%, which is much higher than most of the previously reported metal-free RTP phosphors of CDs in various matrices^18–32,38–43^ and organic RTP materials^1–15,43,48–51^ (Table S2). The high PQE may also be due to the special MCE. A highly rigid environment constructed by the Si-O network plays a significant role in isolating CDs from external quenching factors. Meanwhile, the stable interaction between Si and O network and CDs benefits to minimize the non-radiative decay of long-lived triplets. It is worth mentioning that the PQE gradually decreases as the calcination temperature increases, which is probably owing to the influence of temperature on MCE and the reduction of CDs. When the calcination temperature reaches 500 and 600 °C, the quantum efficiency can reach high values of 26.36% and 21.30%, respectively. It can be observed from the TGA thermogram (Supplementary Fig. 11) that the content of CDs barely decreases between 500 and 600 °C, and thus the main reason for the decrease of PQE should be the change of the spatial confinement structure. It is found that the PQE gradually decreases along with the further increase in temperature due to the reduction of CDs, and thus the quantum efficiency is only 15% at 650 °C. When the temperature gradually increases to 700 °C, the matrix of silica partially collapses and the content of CDs decreases, leading to the decrease of the PQE and phosphorescence emission intensity.

### Stability of the resultant CDs@SiO_2_ phosphors

**Fig. 5:**
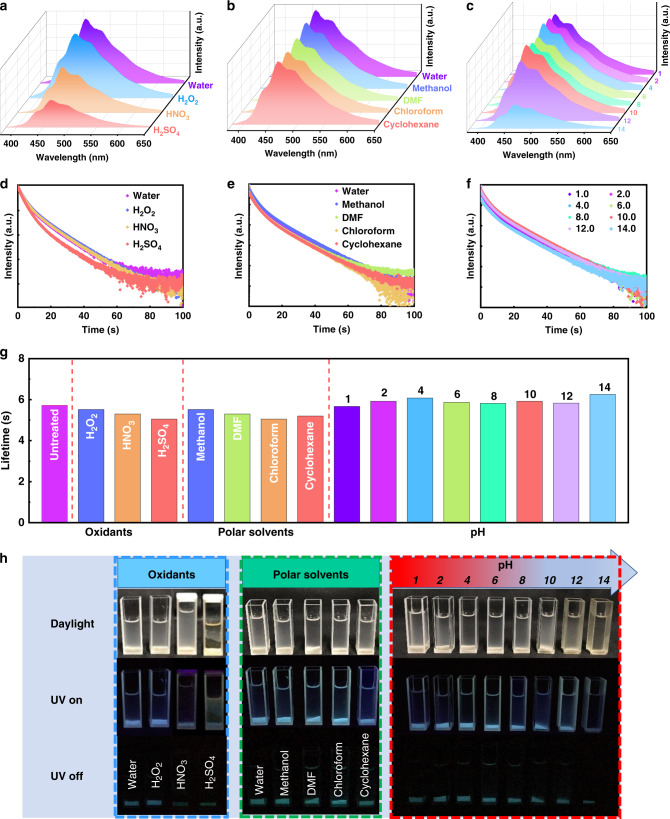
Stability of the resultant CDs@SiO_2_ against strong oxidants, strong acids and bases, water, and polar solvents. **a**, **d** Phosphorescence spectra and time-resolved phosphorescence decay of the untreated CDs@SiO_2_-600 and CDs@SiO_2_-600 oxidized by H_2_O_2_ (30 wt%), concentrated HNO_3_ (30 wt%), or H_2_SO_4_ (98 wt%). **b**, **e** Phosphorescence spectra and time-resolved phosphorescence decay of the CDs@SiO_2_-600 in solvents with different polarities. **c**, **f** Phosphorescence spectra and time-resolved phosphorescence decay of the CDs@SiO_2_-600 under pH values from 1.0 to 14.0. **g** Phosphorescent lifetime histogram of CDs@SiO_2_-600 in various oxidants, polar solvents, and pH values from 1.0 to 14.0. **h** Corresponding photographs of CDs@SiO_2_-600 in various oxidants, polar solvents, and water at different pH under sunlight, UV on and off.

Thanks to the protection by Si–O network, the CDs@SiO_2_ phosphors exhibit excellent anti-quenching properties and exceptional stability. As shown in Fig. 5a, the as-prepared CDs@SiO_2_ powders were first dispersed in strong oxidants, including H_2_O_2_ (30 wt%), concentrated HNO_3_ (30 wt%), and H_2_SO_4_ (98 wt%), and then ultrasonicated for 30 min. It is surprising that the PL is not quenched after being treated by strong oxidants, though the phosphorescence lifetime was slightly decreased, but a very long lifetime afterglow can still be observed with the naked eye (Fig. 5d, h). In order to further investigate the extraordinary stability of the CDs@SiO_2_ phosphors, they were also dispersed in different polar solvents and treated in a similar way. It was found that the phosphors also maintained superior stability in organic polar solvents (Fig. 5b, e, g). To further highlight the remarkable stability, the CDs@SiO_2_ phosphors were similarly treated in aqueous solutions at pH values varying from 1.0 to 14.0. As shown in Fig. 5c and Supplementary Fig. 14, the phosphorescence emission intensity only changed slightly in the pH range of 1.0 to 12.0, which indicates that the CDs@SiO_2_ phosphors can resist strong acids and strong bases. It is known that a concentrated base can corrode silica and thereafter potentially deteriorate the phosphorescence intensity. However, even after being treated by a strong base with a pH of 14.0, the afterglow of the CDs@SiO_2_ phosphors still remains basically unchanged while only the phosphorescence intensity decreases (Fig. 5f–h), further highlighting the protective effect of the Si–O network on the triplet state of CDs. These results indicate that the fabricated CDs@SiO_2_ phosphors manifest an exceptional stability, thanks to the protection by the SiO_2_ network to prevent the quenching of the CDs, making the CDs@SiO_2_ phosphors suitable for various applications especially in harsh environments.

### Universality of the design principles

The above experimental results demonstrate that this work provides a facile and cost-effective method to fabricate metal-free CDs@SiO_2_ phosphors with ultralong lifetime, high phosphorescence efficiency, and exceptional stability from RH biomass. More importantly, this method can be easily expanded to other silicon-rich biomasses, such as wheat husks (WHs) and *Indocalamus* leaves (ILs). As shown in Supplementary Fig. 15, the as-prepared WH-derived and IL-derived phosphors also exhibit ultralong RTP lifetime of 5.31 s and 3.95, respectively. We propose that the cause of this ultralong RTP is similar to RH-derived CDs@SiO_2_ due to the analogous structure and chemical component between RHs and WHs, which is attributed to the MCE of the interstices between Si and O tetrahedrons that can exert a spatial restriction to effectively rigidify the triplet excitons of CDs. These results demonstrate that the present work provides a universal green and low-cost approach to fabricate efficient metal-free RTP materials from natural biomass.

Since the successful achievement of efficient RTP from various silica-based biowaste, we propose that it is possible to achieve efficient RTP by incorporating CDs into the Si–O network using general chemical reagents as starting materials. To further verify our hypothesis and universality of this strategy for fabrication of efficient multi-confined RTP materials, CD1/SiO_2_, CD2/SiO_2_, and CD3/SiO_2_ composites were fabricated by incorporating the three previously reported CDs into SiO_2_ by using Na_2_SiO_3_ and glacial acetic acid as precursors via a sol–gel process similar to CDs@SiO_2_ (please see the Supplementary methods for the detailed experimental procedures). As shown in Supplementary Fig. 16, CD1/SiO_2_, CD2/SiO_2_, and CD3/SiO_2_ composites all exhibit blue-greenish RTP with lifetime of 4.92, 5.25, and 2.79 s, respectively, indicating the feasibility of this MCE to rigidify and suppress the intramolecular vibration of CDs. The success to prepare CD/SiO_2_ with efficient phosphorescence and exceptional stability using regular chemicals (instead of RHs biomass only) ensures the high quality, universality, and reproducibility for potential future large-scale production of multi-confined RTP materials.

### Luminous mechanism of CDs@SiO_2_

Based on the above results, a possible mechanism for realizing such ultralong lifetime and ultrahigh quantum efficiency multi-confined RTP CDs@SiO_2_ with high stability is clearly proved. The CDs first in situ fabricated and incorporated into the vast silicic acid network, and then the calcination process converts such framework into a compact 3D network interconnected by Si–O tetrahedrons. The rigid network can isolate CDs from external quenchers, the stable covalent and hydrogen bonds between the rigid structure and CDs can stabilize the triplet excited states of CDs. More importantly, the sufficient 3D nano-space surrounded by Si–O tetrahedrons exerts spatial restriction that can suppress the intramolecular vibration and thus effectively stabilizing the triplet excited states of the CDs. In addition, it also endows CDs@SiO_2_ with extraordinary stability against oxidants, strong acid and base, and others. This MCE is responsible for this ultralong lifetime and ultrahigh PQE, very different from the previously reported single hydrogen bonds, covalent bonds, or two-dimensional confined interface^18,22,24,28^.

### Time-resolved anti-counterfeiting applications

**Fig. 6:**
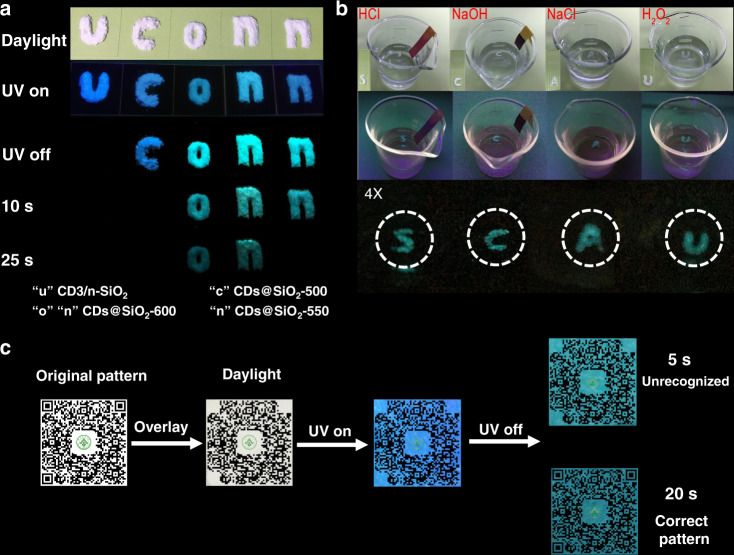
Time-resolved anti-counterfeiting application of CDs@SiO_2_. **a** Digital photographs of information encryption made from CDs/n-SiO_2_, CDs@SiO_2_-500, CDs@SiO_2_-600, and CDs@SiO_2_-550. **b** Digital photographs of phosphorescence from CDs@SiO_2_-600 in different environmental liquids. **c** Digital photographs of information encryption made from CDs@SiO_2_-600 and CDs@SiO_2_-550. The white area was overlaid with CDs@SiO_2_-600, and the black part of the corner is covered with CDs@SiO_2_-550.

Given the ultralong phosphorescence emission up to 40 s to the naked eye and high stability, the CDs@SiO_2_ phosphors can be employed as an ideal material for use in time-resolved anti-counterfeiting, because such a long time of phosphorescence is very rare and hard to duplicate. As shown in Fig. 6a, we simply designed an alphabetic security code by using CDs/*n*-SiO_2_, CDs@SiO_2_-500, CDs@SiO_2_-600, and CDs@SiO_2_-550. Under the UV irradiation, the white pattern of “uconn” in daylight exhibit blue FL. Once stopping the excitation, because of the different phosphorescence emission time from CDs@SiO_2_ and free phosphorescence observed from CDs/*n*-SiO_2_, only “conn” was shown. Furthermore, as time goes on to 10 and 25 s, the “c” and the last “n” gradually disappeared, respectively. The encrypted pattern “on” was finally left behind after a layer of time screening (more than 25 s). These results indicate the great promise of the as-fabricated CDs@SiO_2_ phosphors with ultralong lifetime for potential applications in the field of time-resolved encryption.

Figure 6b shows the patterns of “S” “C” “A” “U” immersed in different environmental liquids including HCl (2 M), NaOH (2 M), NaCl (2 M), and H_2_O_2_ (30%). Lasting blue-greenish phosphorescence after stopping the excitation can be observed, suggesting that the CDs@SiO_2_ can be used in various harsh environments. To prove the performance of the CDs@SiO_2_ as a unique anti-counterfeiting tool, a QR (quick response) code with implicit information was also fabricated. As illustrated in Fig. 6c, the gray part of the pattern was overlaid with CDs@SiO_2_-600 powders. To more distinctly compare the phosphorescence, CDs@SiO_2_-550 was also employed to cover three black corners of the code. Under the daylight or a 254 nm UV lamp, the QR code cannot be identified to provide any information because the identification division was partially covered. Furthermore, it is interesting to observe that the phosphorescence from covering the corner with CDs@SiO_2_-550 has already quenched while the gray part overlaid with CDs@SiO_2_-600 is still obvious 20 s later. Therefore, when the UV lamp was switched off, an unrecognizable incomplete pattern was first obtained. Then the black part of the corner gradually appears along the time, and eventually a correct blue-greenish pattern is visible after 20 s, which could be recognized by a scanner. The as-prepared special multi-confined RTP CDs@SiO_2_ phosphors with ultralong lifetime, ultrahigh quantum efficiency, and high stability are also expected to find applications in biomedical, optoelectronic, etc.

## Discussion

In summary, a room-temperature phosphorescence material of multi-confined CDs simultaneously with ultralong lifetime, high quantum efficiency, and excellent stability is designed and fabricated by a universal strategy. An ultralong RTP emission with lifetime up to 5.72 s (more than 40 s to the naked eye) and high PQE of 21.30% can be simultaneously achieved through MCE originated from Si to O network. The maximum absolute PQE of the resultant phosphors can reach 26.36%. Furthermore, the multi-confined CDs@SiO_2_ phosphors show exceptional stability in strong oxidant agents (i.e., H_2_O_2_, concentrated HNO_3_, and H_2_SO_4_), organic solvents with different polarities, strong acids, and strong bases. The experiment results suggest that the MCE of highly rigid network, stable covalent/hydrogen bonds, and sufficient 3D nano-space is responsible for this ultralong lifetime, high quantum efficiency, and extraordinary stability of the as-designed CD-based multi-confined RTP material. We later verified that multi-confined RTP materials can be synthesized using regular chemicals via this universal strategy. The findings offer design principles and insights to fabricate metal-free RTP materials for promising applications in biomedicine, optical anti-counterfeiting, and optoelectronics, especially for applications under extreme environments.

## Methods

### Materials

The RHs were collected from a mill in Guangzhou, China. Glacial acetic acid (HAc) and hydrochloric acid (HCl) were purchased from Guangzhou Chemical Reagent Co., Ltd., and sodium hydroxide (NaOH) was purchased from Guangdong Guanghua Sci-Tech Co., Ltd. Deionized water used in this project was supplied by a Water Purifier Nano pure water system (Master-E, Hitech-Science tool, Shanghai, China). All reagents are of analytical grade and were used without further purification.

### Synthesis of CDs@SiO_2_ RTP materials

In a typical process, 10 g of dried RHs were first ground into fine powders (200 mesh), soaked with a HCl solution (2.0 M) for 2 h under magnetic stirring, and then washed to neutral with deionized water. Subsequently, the HCl pretreated RHs were refluxed in 100 mL of NaOH solution (0.8 M) in a round bottom flask with magnetic stirring at 160 °C for 6 h. Then, the mixture was filtered to obtain the CD-containing mother liquor. HAc was added drop-wise into the aforementioned mother liquor under magnetic stirring, until the pH reached 5–6 to ensure proper gelation. The as-resulted solution was then aged for 6 h to form a chocolate-brown solid gel, which was then washed with deionized water and ethanol to remove surface-attached CDs and inorganic salts. After that, the gel was dried at 60 °C in a vacuum oven for 10 h and ground into fine powders for further use.

The as-prepared gel powders were calcined in air in a furnace with a ramp rate of 5 °C/min to obtain CDs@SiO_2_ composites. During the calcination process, the optimum processing condition was determined by calcining various temperatures in the range of 400–700 °C for 90 min. Hereinto, the samples were denoted as CDs@SiO_2_-*x*, where *x* refers to the calcination temperature.

### Characterization

UV–vis absorption spectra of the samples were recorded on a Shimadzu UV-2550 ultraviolet-visible spectrophotometer. XRD (Rigaku) was conducted in the 2*θ* range from 10° to 80°. Infrared spectra were acquired using a Nicolet Avatar 360 FTIR spectrophotometer from 500 to 4000 cm^−1^ wavenumber. HRTEM (JEOL-2010) images were collected to characterize the structure and morphology of the samples. PL spectra and time-resolved decay curves were measured on a fluorescence spectrophotometer (Hitachi Model F-7000) equipped with a 150 W Xenon lamp as the excitation source. The phosphorescence quantum yields were measured by an Edinburgh FLS920 spectrophotometer with an integrating sphere. The parameters are listed as follows: a microsecond pulse lamp light source; excitation wavelength: 260 nm; sample window: 50 ms; excitation period: 60 ms; delay after excitation: 0.1 ms; step size: 1 nm; dwell time: 0.1 s. And then the quantum yield was calculated after measuring the excitation area and emission peak area of the background and the samples. Thermogravimetric analysis (TGA, recorded by a TG-DSC system, Netzsch) was conducted from room temperature to 1000 °C at a rate of 10 °C/min in an air atmosphere. XPS experiments were performed using a Thermo Fisher 250Xi X-ray photoelectron spectrometer with a monochromatic Al Kα X-ray source. Electron spin-resonance spectroscopy was recorded on an electron spin resonance instrument (JES FA200, JEOL, Japan).

## Supplementary information

Supplementary Information

Supplementary Movie

Description of Additional Supplementary Files

## Data Availability

The data that supports the findings of this study are available from the corresponding authors upon request.
