# Supplementary material for: Ultralong lifetime and efficient room temperature phosphorescent carbon dots through multi-confinement structure design
Source: Nat Commun. 2020 Nov 5;11:5591. doi: 10.1038/s41467-020-19422-4 (PMC7645781; doi:10.1038/s41467-020-19422-4)
Supplement: Supplementary file 1 — Supplementary Information [file 41467_2020_19422_MOESM1_ESM.pdf]

Supplementary Information

Ultralong lifetime and efficient room temperature phosphorescent carbon dots through multi-confinement structure design

Sun et al

Supplementary Methods

Synthesis of CDs/n-SiO_2 nanocomposite. Nano SiO_2 sol (n-SiO_2) was prepared by mixing TEOS, ethanol, H_2O, and HCl (pH= 2) in a molar ratio of 1:4:5: 0.2 followed by refluxing at 80 °C for 120 min.[1] A dispersion containing RH-derived CDs (2 ml) was then added into the above n-SiO_2 (20 ml) and kept under stirring at room temperature until a uniform gel formed. The dried gel powders were then calcinated at 600 °C for 90 min to obtain the final CDs/n-SiO_2 nanocomposite.

Synthesis of carbon dots 1 (CD1) and CD1/SiO_2. CD1 was prepared according to a reported method with some modifications.[2] A sample of 2 g of citric acid (CA) was dissolved in 10 mL deionized water and then heated in a conventional microwave oven for 7 min. After the reaction, the sample was cooled to room temperature naturally. The cluster-like product was re-dissolved in 10 mL deionized water and subjected to dialysis in deionized water to obtain CD1. CD1/SiO_2 was prepared by mixing 10 mg CD1 and 1 g of Na_2SiO_3 in 20 mL deionized water, then glacial acetic acid was added drop-wise into the above solution until the pH reached 5-6 to ensure proper gelation. The gel was then washed with deionized water and ethanol to remove surface-attached CDs and inorganic salts. After that, the gel was dried at 60 °C for 10 h and ground into fine powders. CD1/SiO_2 was obtained after calcining the powders at 600 °C for 90 min.

Synthesis of carbon dots 2 (CD2) and CD2/SiO_2. CD2 was prepared using a hydrothermal method according to a reported method.[3] A sample of 2 g of polyvinyl alcohol (PVA) was dissolved in deionized water (40 mL) at 80 °C before mixing with ethylenediamine (EDA, 1.5

mL). Then, the mixture was delivered to a Teflon autoclave (volume of 100 mL) for a hydrothermal treatment at 220 °C for 10 h. After reaction, acetone was introduced to precipitate excessive PVA. After centrifugation process, the transparent solution was dialysis (MWCO = 1000 Da) and freeze dried to obtain CD2. CD2/SiO_2 was prepared via the same method to prepare CD1/SiO_2 as detailed above.

Synthesis of carbon dots 3 (CD3) and CD3/SiO_2. CD3 was prepared in accordance with the reported procedures.[4] Briefly, 1 g of CA was dissolved in 30 mL of deionized water before mixing with EDA (1.6 mL). The mixture was then heated at 200 °C for 5 h via a hydrothermal method and then cooled down to room temperature. The obtained yellowish-brown solution was dialysis (MWCO = 1000 Da) and freeze dried to obtain CD3. The CD3/SiO_2 was prepared via the same method to produce CD1/SiO_2 as detailed above.

Supplementary Figures

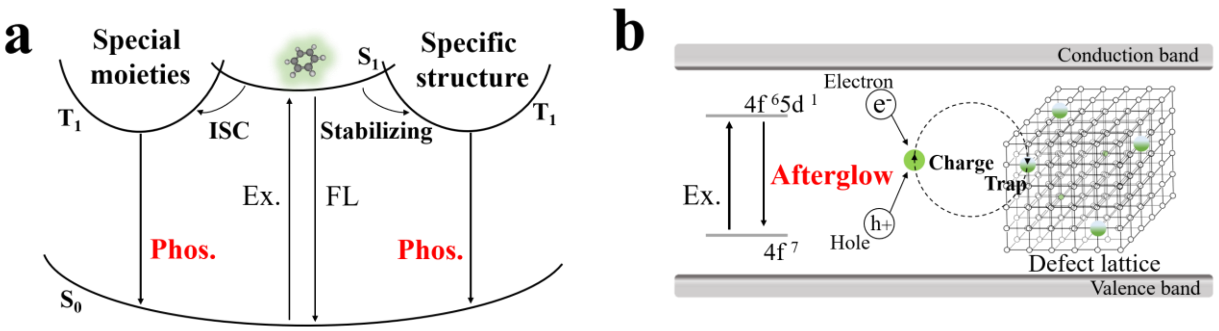

Supplementary Figure 1. (a) Typical mechanism of organic phosphorescence by forming specific structure or adding special moieties. (S_0: ground state; S_1: first excited singlet state; T_1: first excited triplet state; Ex: Excitation, FL: Fluorescence, Phos: Phosphorescence, ISC: Intersystem crossing) (b) Typical mechanism of inorganic afterglow materials.

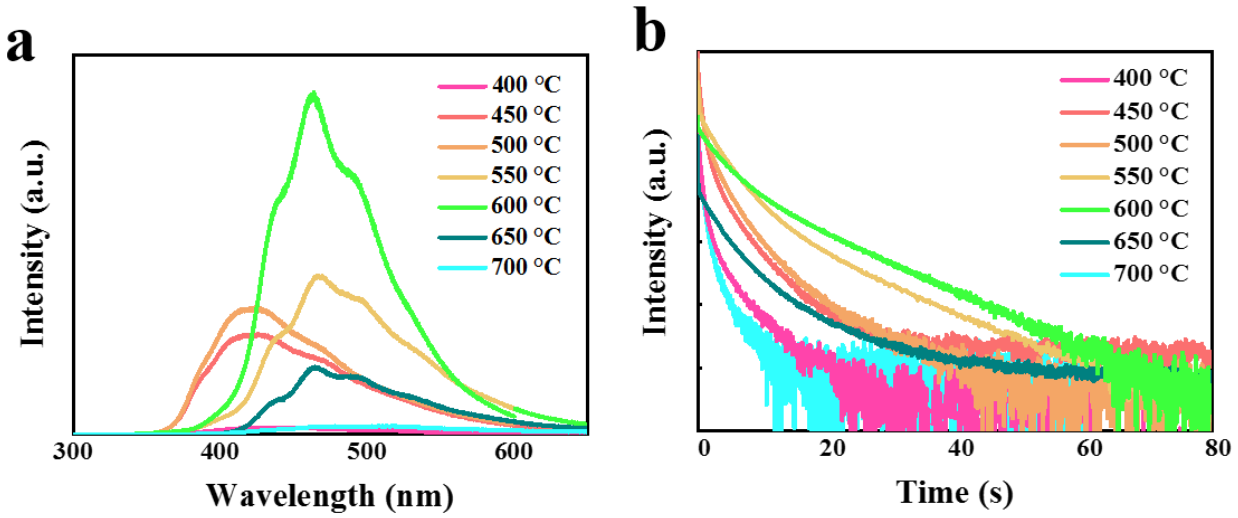

Supplementary Figure 2. Phosphorescence spectra (a) and time-resolved phosphorescence decay spectra (b) of the CDs@SiO_2 phosphors calcined at temperatures ranging from 400 to 700 °C.

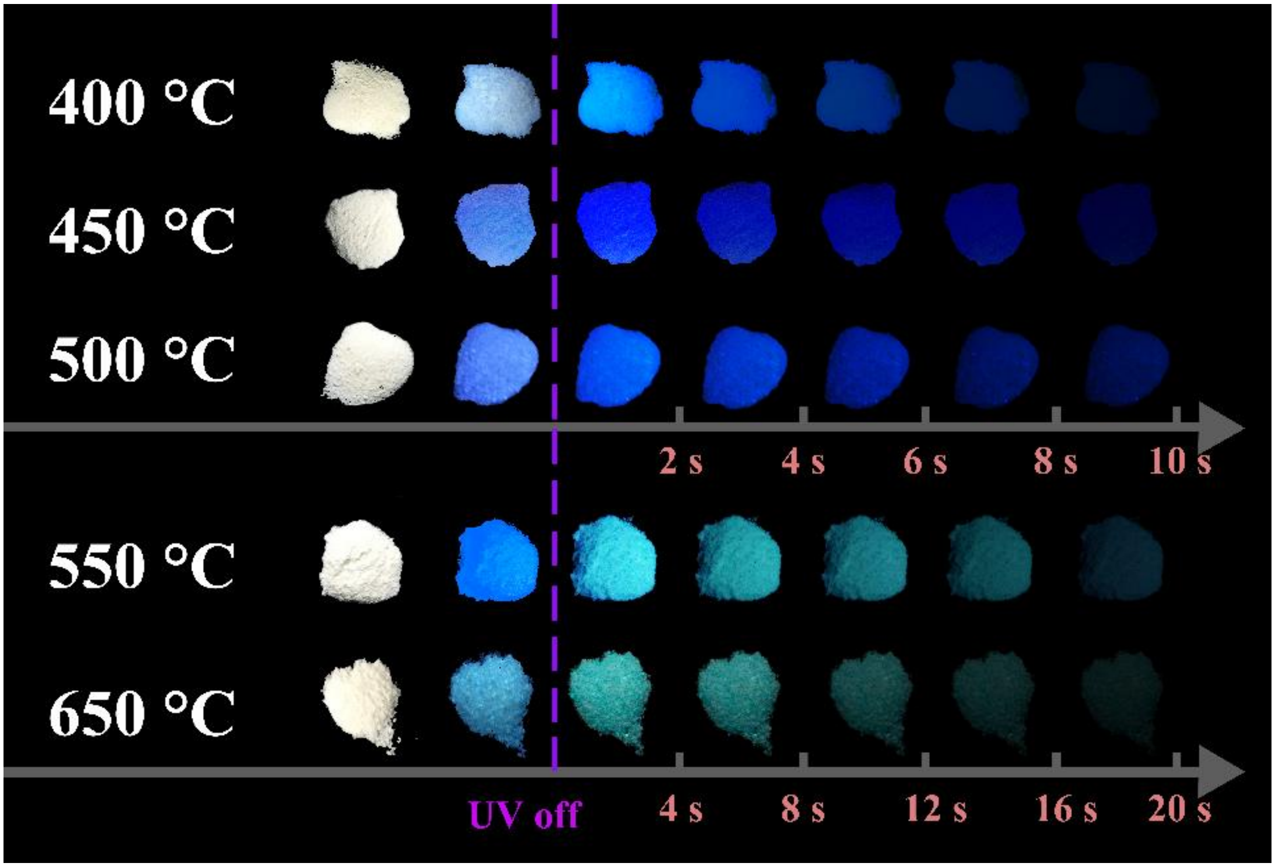

Supplementary Figure 3. Photographs in daylight, UV light, and different delay time after UV irradiation of the CDs@SiO_2 phosphors calcined at different temperatures.

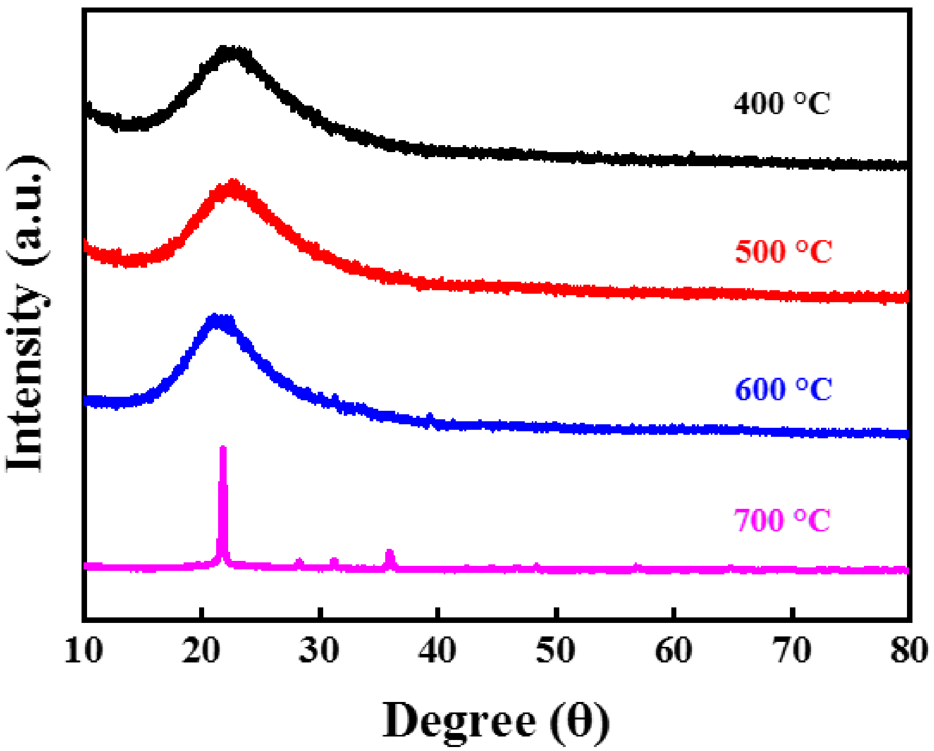

Supplementary Figure 4. XRD pattern of CDs@SiO_2-600.

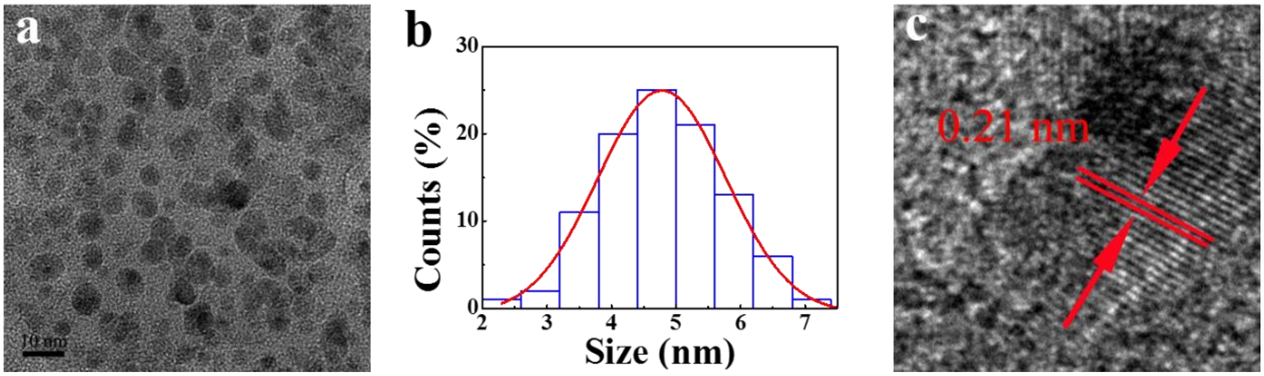

Supplementary Figure 5. (a) TEM image, (b) size distribution histogram, and (c) HRTEM image of the pristine RH-derived CDs.

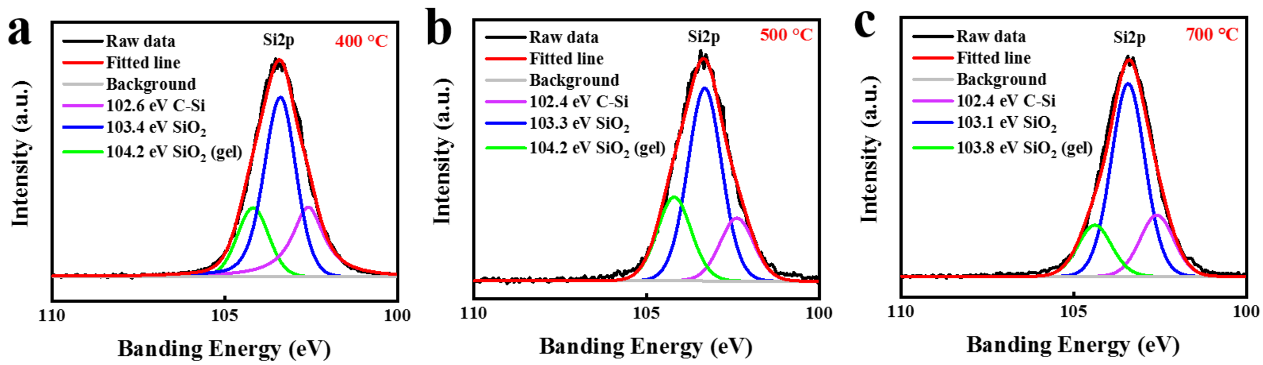

Supplementary Figure 6. High resolution XPS spectrum and fitting results of Si 2p in CDs@SiO_2 phosphors calcined at 400 °C (a), 500 °C (b) and 700 °C (c), respectively.

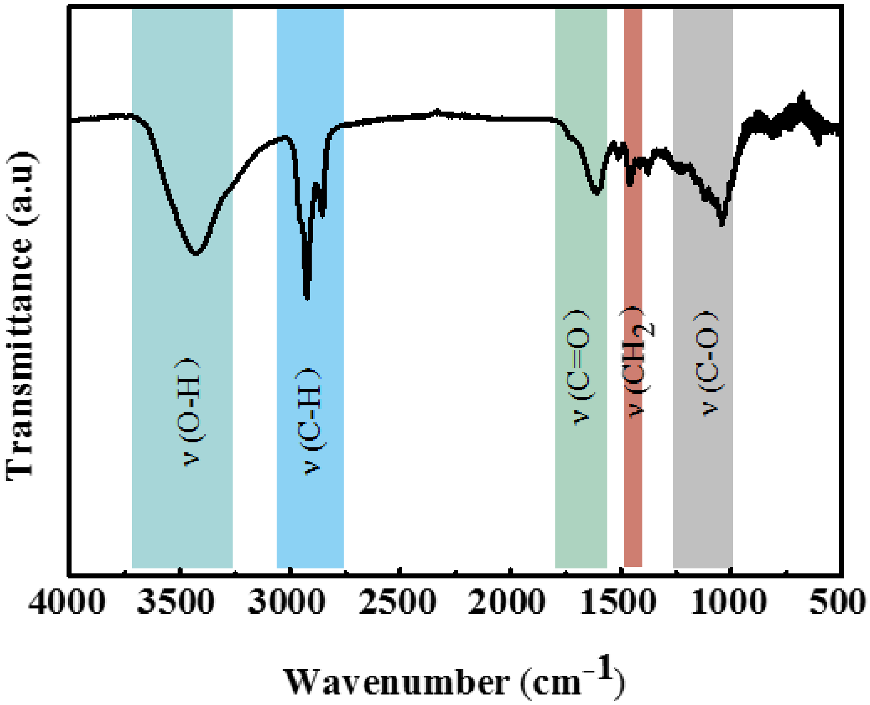

Supplementary Figure 7. FTIR spectrum of the pristine RH-derived CDs.

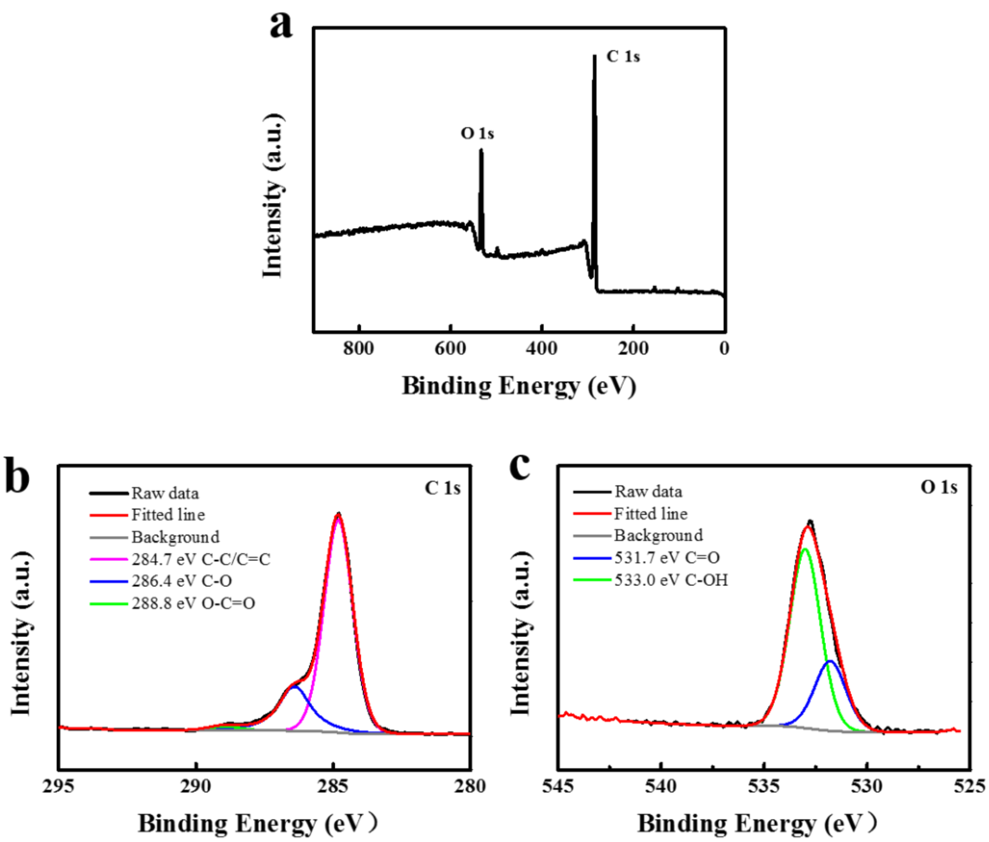

Supplementary Figure 8. XPS spectra of the pristine RH-derived CDs.

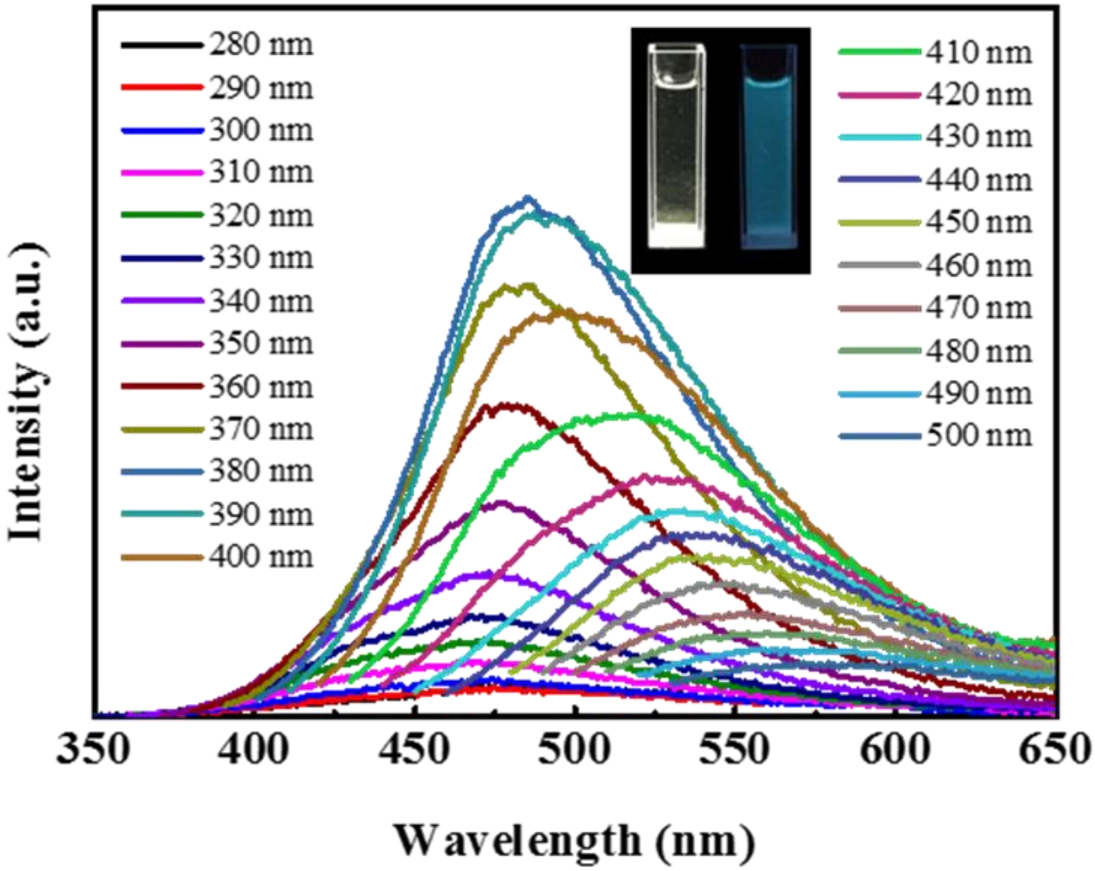

Supplementary Figure 9. FL spectra of the pristine RH-derived CDs.

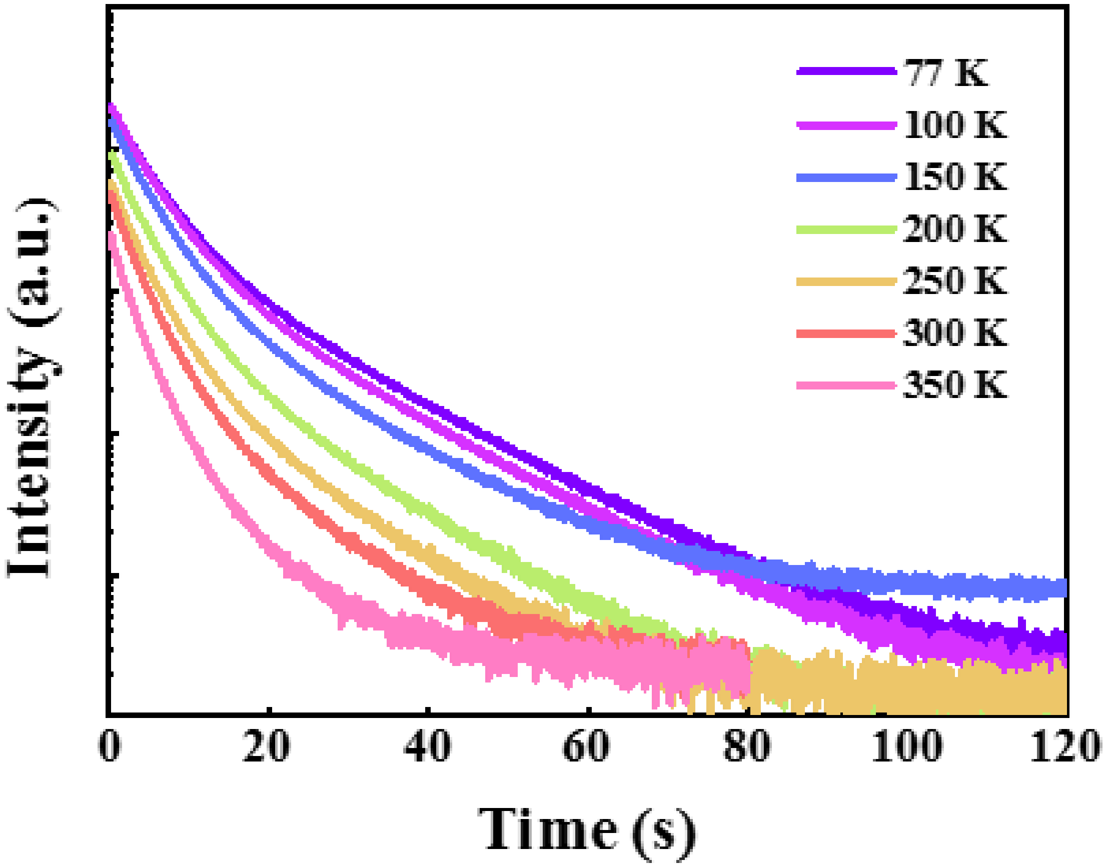

Supplementary Figure 10. Phosphorescence decay spectra of CDs@SiO_2-600 phosphor tested at different temperatures under 260 nm excitation.

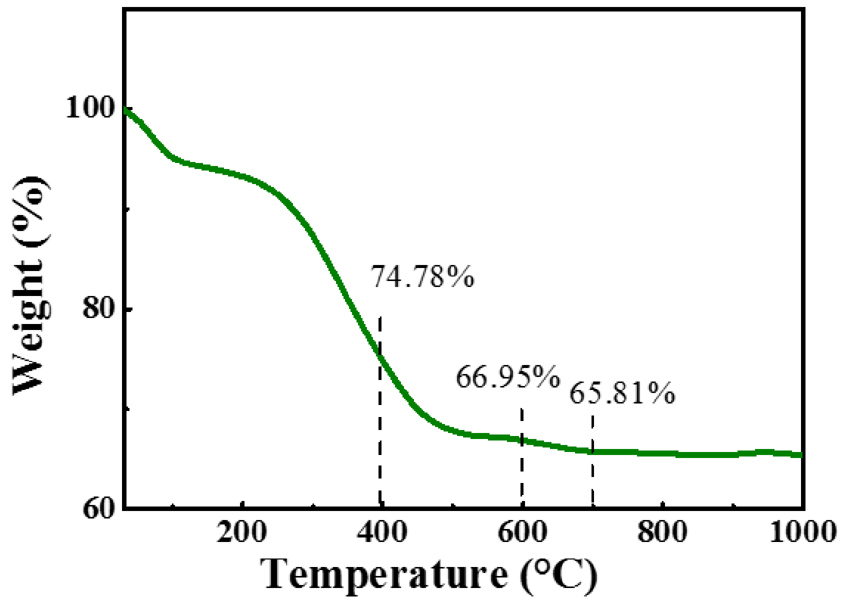

Supplementary Figure 11. TGA thermogram of the gel precursor.

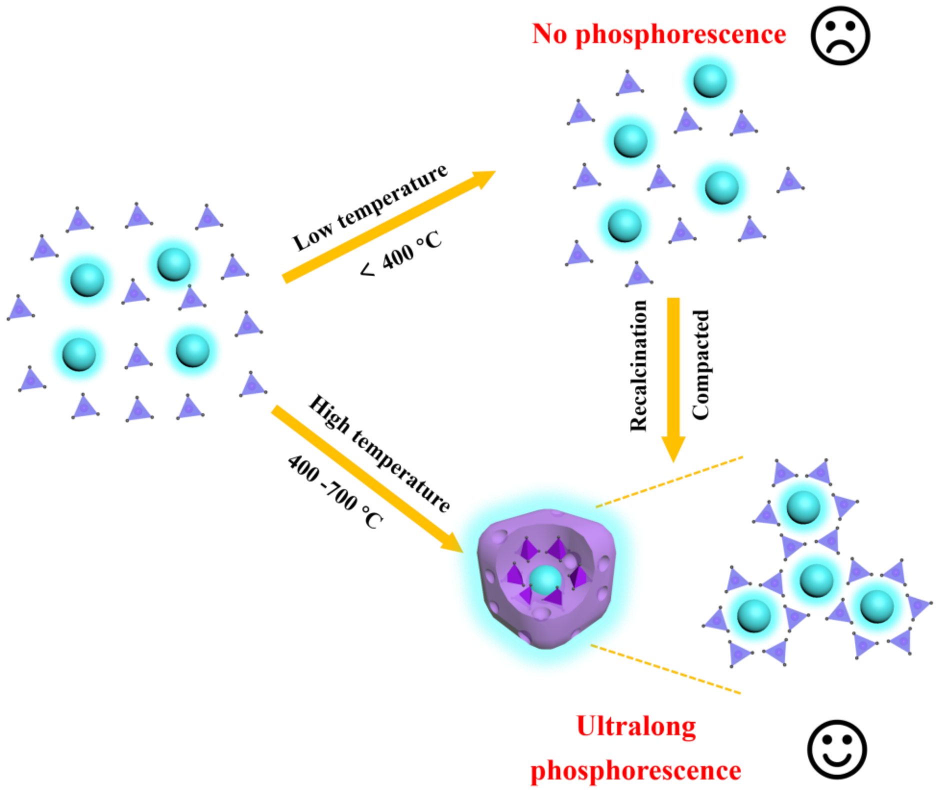

Supplementary Figure 12. Schematic of the effect of temperature for fabrication of the metal-free ultralong phosphorescence CDs@SiO_2 materials.

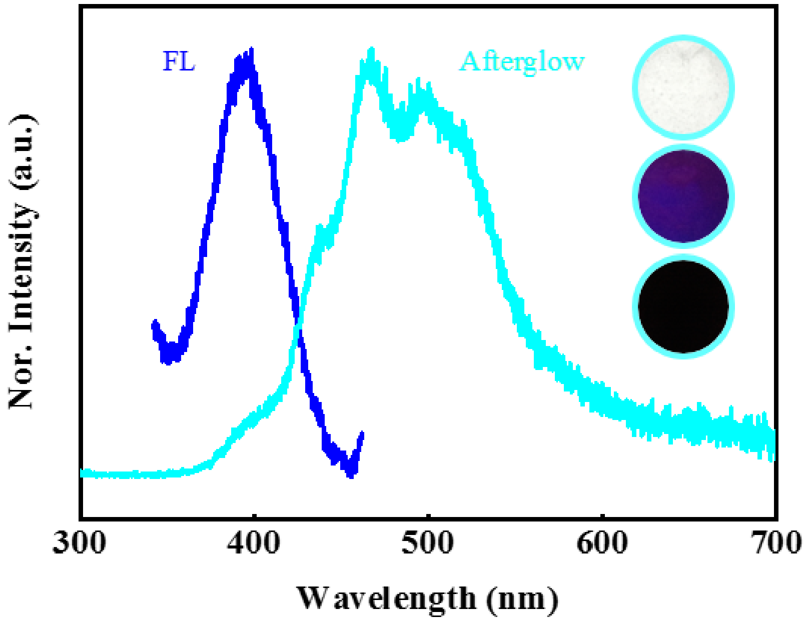

Supplementary Figure 13. Fluorescence and phosphorescence spectra of the comparative CDs/n-SiO_2/ nanocomposites. The inset shows the photographs of the comparative CDs/n-SiO_2 nanocomposite under sunlight (top), UV (middle), and after switching off UV (bottom).

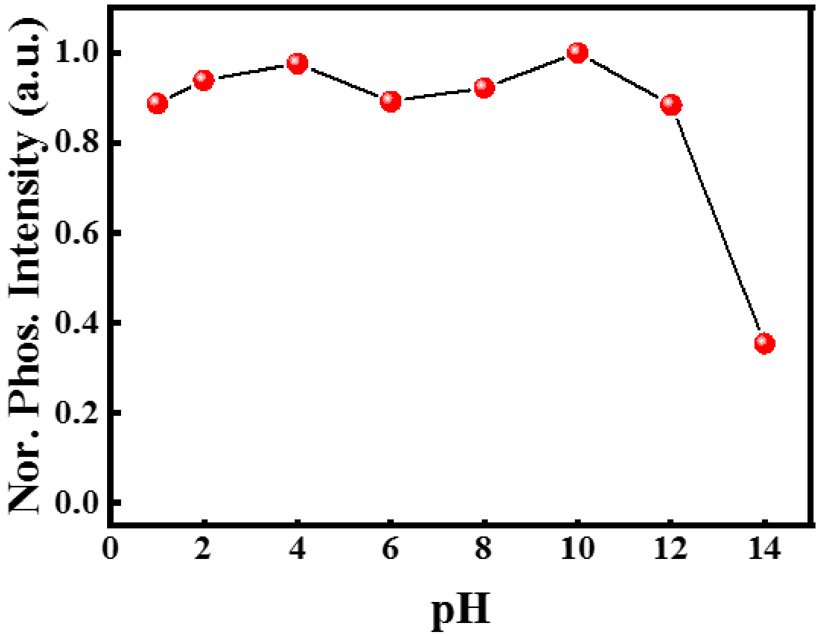

Supplementary Figure 14. Comparison of the phosphorescence intensity of CDs@SiO_2 under different pH values.

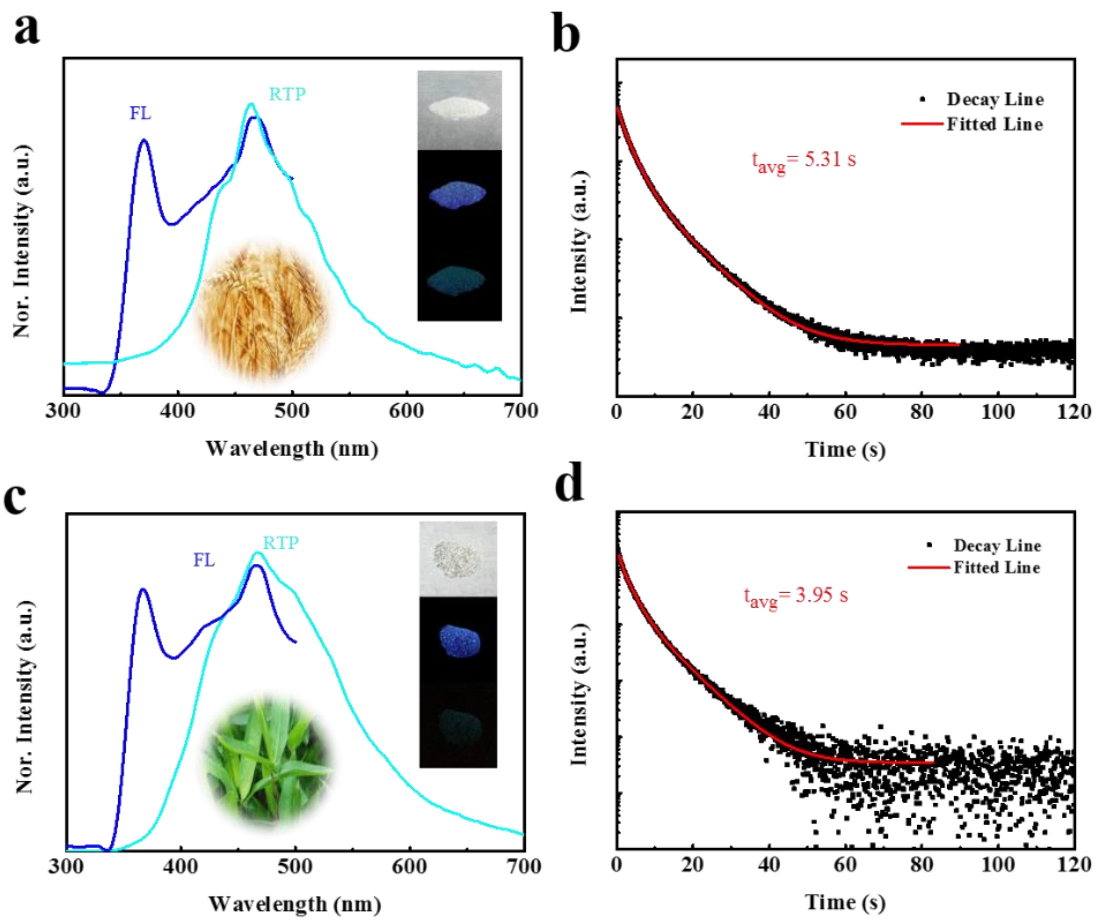

Supplementary Figure 15. (a) Fluorescence and phosphorescence spectra, photograph under sunlight, UV, and after UV turned off (inset) of wheat husk (WH)-derived RTP materials. (b) Time-resolved phosphorescence decay of WH-derived RTP materials. (c) Fluorescence and phosphorescence spectra, photograph under sunlight, UV, and after UV turned off (inset) of *Indocalamus* leave (IL)-derived RTP materials. (d) Time-resolved phosphorescence decay of IL-derived RTP materials.

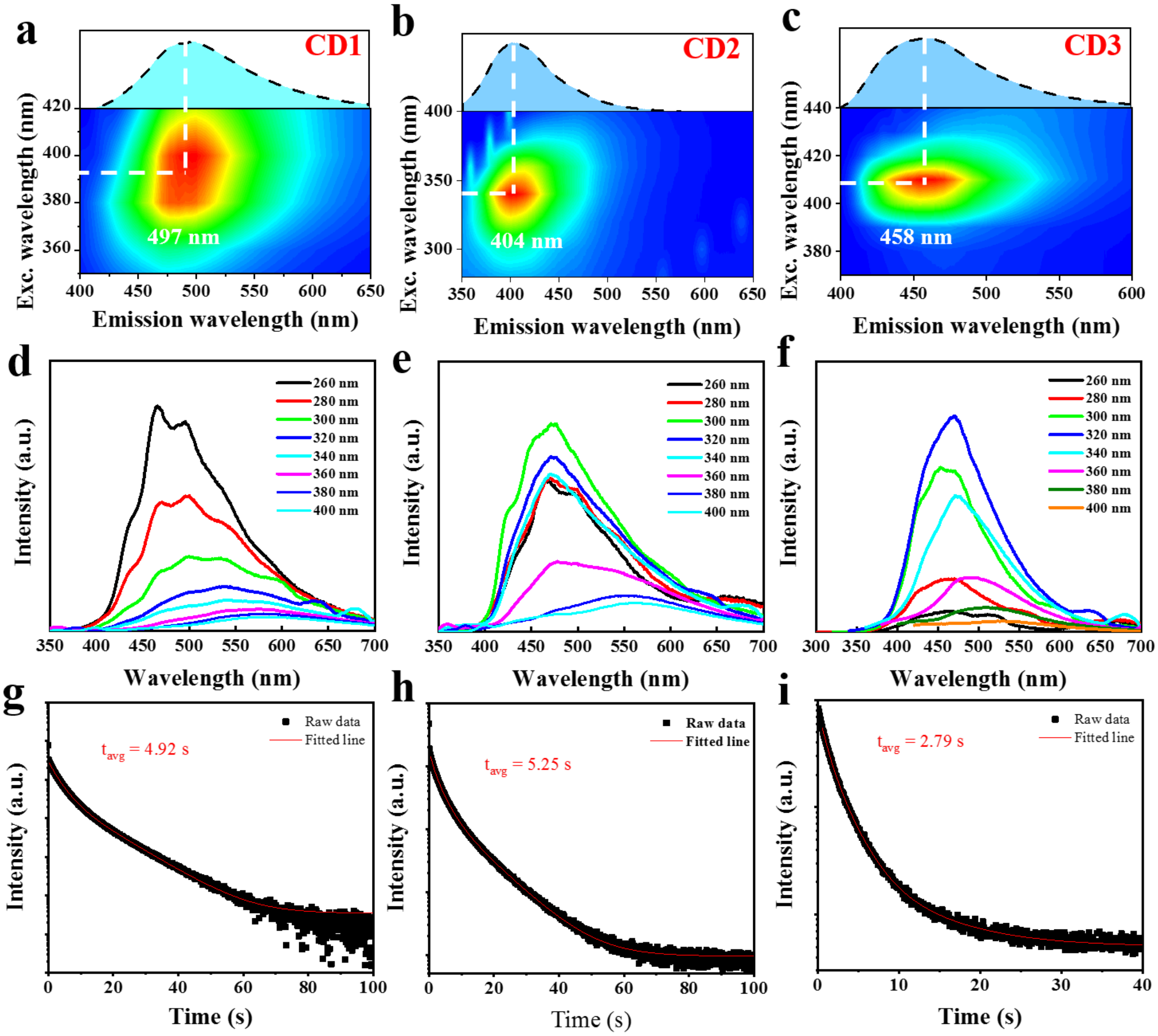

Supplementary Figure 16. (a-c) FL spectra of CD1, CD2 and CD3, respectively. (d-f) Phosphorescence spectra of CD1/SiO_2, CD2/SiO_2, and CD3/SiO_2, respectively. (g-i) Time-resolved phosphorescence decay of CD1/SiO_2, CD2/SiO_2, and CD3/SiO_2, respectively.

Supplementary Tables

Supplementary Table 1. Phosphorescence lifetime of the CDs@SiO_2 phosphors calcined at temperatures ranging from 400 to 700 °C.

Sample	τ_1 (s)	B_1 (%)	τ_2 (s)	B_2 (%)	τ_3 (s)	B_3 (%)	τ_{avg} (s)
**400 °C**	3.22119	2.64	0.69458	25.71	0.26035	71.65	**0.99**
**450 °C**	3.04342	3.63	0.78804	27.71	0.32246	68.66	**1.05**
**500 °C**	1.70267	26.63	4.34034	4.08	0.53491	69.29	**1.74**
**550 °C**	2.98075	55.69	1.46992	39.65	9.21216	4.66	**3.65**
**600 °C**	9.53805	17.10	3.23189	56.87	1.53309	26.03	**5.72**
**650 °C**	6.7762	13.63	2.28087	77.80	0.35513	8.57	**3.78**
**700 °C**	1.40049	4.86	0.36102	58.69	0.11813	36.45	**0.55**

Supplementary Table 2. Comparison of the phosphorescence lifetime and absolute phosphorescence quantum efficiency (PQE) with the previously reported CDs-based and organic RTP materials.

Matrix	τ_{avg}	PQE	Reference
**PVA**	0.45 s	-	5
**CA**	0.687 s	-	6
**PVA**	0.572 s	25.57%	7
**PVA**	0.456 s	-	8
**Zeolite**	0.35 s	-	9
**SiO_2**	1.8 s	-	10
**SiO_2**	0.703 s	-	11
**$KAl(SO_4)_2 \cdot 12H_2O$**	0.655 s	-	12
**$Al_2(SO_4)_3$**	0.876 s	-	13
**PVA**	0.38 s	-	14
**PVA**	0.707 s	24%	15
**PU**	0.0087 s	-	16
**Urea**	1.06 s	7%	17
**Urea**	1.11 s	-	18
**LDHs**	0.386 s	5.99%	19
**CA**	0.705 s	14%	20
**BA**	2.26 s	17.5%	21
**SiO_2**	1.26 s	-	22
**Zeolite**	0.57 s	-	23
**SiO_2 (500 °C)**	**1.74 s**	**26.36%**	**This work**
**SiO_2 (600 °C)**	**5.72 s**	**21.30%**	**This work**

Supplementary Table 3. Phosphorescence lifetime of CDs@SiO_2-600 phosphor tested at different temperatures.

Temperature	τ_1 (s)	B_1 (%)	τ_2 (s)	B_2 (%)	τ_3 (s)	B_3 (%)	τ_{avg} (s)
**77 K**	1566.419	22.08	306.0661	69.54	260.4767	8.38	**7.45**
**100 K**	258.9197	68.90	1623.046	22.27	288.5112	8.83	**6.76**
**150 K**	212.1957	7.98	1323.400	22.49	189.0511	69.53	**6.10**
**200 K**	794.1080	22.96	127.3645	7.81	102.9989	69.23	**5.33**
**250 K**	478.5379	22.75	61.30262	68.74	99.32996	8.51	**4.90**
**300 K**	402.7036	23.89	73.90298	7.29	58.27715	68.82	**4.14**
**350 K**	193.6204	24.53	49.30653	8.70	25.07259	66.77	**3.43**

Supplementary Movie caption:

Supplementary Movie 1. Afterglow of CDs@SiO_2-600 phosphors immediately after switching off the 254 nm UV light.

Supplementary References:

1. Li, W. et al. Preparation and properties of carbon dot-grafted $CaAl_{12}O_{19}:Mn^{4+}$ color-tunable hybrid phosphor. *Adv. Opt. Mater*. **4**, 427-434 (2016).
2. Dong, Y. et al. Graphene quantum dot as a green and facile sensor for free chlorine in drinking water. *Chen, Anal. Chem*. **84**, 8378-8382 (2012).
3. Chen, Y. et al. A self-quenching-resistant carbon-dot powder with tunable solid-state fluorescence and construction of dual-fluorescence morphologies for white light-emission. *Adv. Mater*. **28**, 312-318 (2016).
4. Zhu, S. et al. Highly photoluminescent carbon dots for multicolor patterning, sensors, and bioimaging. *Angew. Chem. Int. Ed*. **52**, 3953-3957 (2013).
5. Tan, J. et al. Synthesis of amphiphilic carbon quantum dots with phosphorescence properties and their multifunctional applications. *J. Mater. Chem. C*. **4**, 10146-10153 (2016).
6. Li, Q. et al. Induction of long-lived room temperature phosphorescence of carbon dots by water in hydrogen-bonded matrices. *Nat. Commun*. **9**, 734-742 (2018).
7. Xia, C. et al. Hydrothermal addition polymerization for ultrahigh-yield carbonized polymer dots with room temperature phosphorescence via nanocomposite. *Chem. Eur. J.* **24**, 11303-1138 (2018).
8. Jiang, K. et al. Triple-mode emission of carbon dots: applications for advanced anti-counterfeiting. *Angew. Chem. Int. Ed*. **55**, 7231-7235 (2016).
9. Liu, J. et al. Carbon dots in zeolites: A new class of thermally activated delayed

fluorescence materials with ultralong lifetimes. *Sci. Adv*. **3**, e1603171 (2017).

10. Joseph, J. and Anappara, A. Cool white, persistent room-temperature phosphorescence in carbon dots embedded in a silica gel matrix. *Phys. Chem. Chem. Phys*. **19**, 15137-15144 (2017).

11. Jiang, K. et al. Activating room temperature long afterglow of carbon dots via covalent fixation. *Chem. Mater*. **29**, 4866-4873 (2017).

12. Dong, X. et al. Efficient long lifetime room temperature phosphorescence of carbon dots in a potash alum matrix. *J. Mater. Chem. C.* **3**, 2798-2801 (2015).

13. Joseph, J. and Anappara, A. Long life-time room–temperature phosphorescence of carbon dots in aluminum sulfate. *Chem. Select*. **2**, 4058-4062 (2017).

14. Deng, Y. et al. Long lifetime pure organic phosphorescence based on water soluble carbon dots. *Chem. Commun*. **49**, 5751 (2013).

15. Tian, Z. et al. Multilevel data encryption using thermal-treatment controlled room temperature phosphorescence of carbon dot/polyvinylalcohol composites. *Adv. Sci.* 1800795 (2018).

16. Tan, J. et al. Large-scale synthesis of N-doped carbon quantum dots and their phosphorescence properties in a polyurethane matrix. *Nanoscale.* **8**, 4742-4747 (2016).

17. Li, Q. et al. Efficient room-temperature phosphorescence from nitrogen-doped carbon dots in composite matrices. *Chem. Mater*. **28**, 8221-8227 (2016).

18. Lin, C. et al. Blue, green, and red full-color ultralong afterglow in nitrogen doped carbon dots. *Nanoscale.* **11**, 6584-6590 (2019).

19. Bai, L. et al. Activating efficient room temperature phosphorescence of carbon dots by synergism of orderly non-noble metals and dual structural confinements. *Nanoscale.* **9**, 6658-6664 (2017).

20. Tan, J. et al. High pH-induced efficient room-temperature phosphorescence from carbon dots in hydrogen-bonded matrices. *J. Mater. Chem. C.* **6**, 7890-7895 (2018).

21. Li, W. et al. A universal strategy for activating the multi-color room temperature afterglow of carbon dots in a boric acid matrix. *Angew. Chem. Int. Ed*. **58**, 1-7 (2019).

22. Tang, G. et al. One-step preparation of silica microspheres with super-stable ultralong room temperature phosphorescence. *J. Mater. Chem. C.* **7**, 8680-8687 (2019).

23. Liu, J. et al. Template-modulated afterglow of carbon dots in zeolites: room-temperature phosphorescence and thermally activated delayed fluorescence. *ACS Mater. Lett.* **1**, 58-63 (2019).
