# Supplementary material for: Ultralong lifetime and efficient room temperature phosphorescent carbon dots through multi-confinement structure design
Source: Nat Commun. 2020 Nov 5;11:5591. doi: 10.1038/s41467-020-19422-4 (PMC7645781; doi:10.1038/s41467-020-19422-4)
Supplement: Supplementary file 3 — Description of Additional Supplementary Files [file 41467_2020_19422_MOESM3_ESM.pdf]

**Title: Supplementary Movie 1.**

**Description:** Afterglow of CDs@$SiO_2$-600 phosphors immediately after switching off the 254 nm UV light.
